# Anterolateral Thigh Flap Reconstruction of Full Thickness Lateral Abdominal Wall Defect from Desmoid Tumour

**DOI:** 10.1155/2024/1908212

**Published:** 2024-01-16

**Authors:** Melissa Yeo

**Affiliations:** Department of General Surgery, Singapore General Hospital, Singapore, Singapore

## Abstract

Desmoid tumours are benign but locally aggressive mesenchymal neoplasms that occur most commonly in the abdomen, with the potential to invade surrounding structures causing significant morbidity. Lateral abdominal wall defects are known to be more challenging and less frequently encountered compared to ventral abdominal wall defects. Asymmetric forces caused by contraction of remnant rectus and contralateral oblique muscles increase the risk of herniation postoperatively. We report a case of a challenging abdominal wall reconstruction after desmoid tumour resection in a 62-year-old male patient who presented to our hospital with a progressively enlarging left upper back lump of 6 months duration. A venous supercharged pedicled anterolateral thigh flap was combined with PROLENE® mesh for reconstruction, and the patient recovered well with good functional and aesthetic outcomes at 2-year follow-up. The pedicled anterolateral thigh flap with venous supercharging can be effectively used for the reconstruction of extensive lateral abdominal wall defects.

## 1. Introduction

The abdominal wall has vital static and dynamic functions. Static functions include housing the internal organs as well as supporting an erect posture. Dynamic functions are mainly in generating Valsava for urinating, coughing, and bowel movement. Anterior abdominal wall defects have been the focus of abdominal wall reconstruction as they are much more common than lateral abdominal wall defects. Incisional hernia following exploratory laparotomy occurs in 5-20% of patients [1], and parastomal defects are usually located in the anterior abdominal wall. Conversely, lateral abdominal wall defects are usually traumatic in origin or from the resection of tumours. Anterior and lateral abdominal wall defects have clear anatomical and biomechanical distinctions. The anterior abdominal wall consists of the paired rectus abdominis muscles within the rectus sheath separated by the linea alba and its main function is in trunk flexion. The lateral abdominal wall extends horizontally from the linea semilunaris to the posterior paraspinal muscles and vertically from the costal margin to the iliac crest. It is formed by the decussating fibres of the external oblique, internal oblique, and transversus abdominis muscles. Baumann and Butler [2] opined that lateral defects may be further subdivided by anatomic region into paramedian, lateral, subcostal, and paraspinal defects. Paramedian defects affect the linea semilunaris but spare the linea alba. Lateral defects involve the lateral abdominal muscles between the costal margin and the iliac crest. Subcostal defects involve the upper abdomen, chest wall, and diaphragm, whereas paraspinal defects are lumbar defects that involve the retroperitoneum and the posterior chest wall. Lateral abdominal wall defects therefore present a much larger potential surface area that can be lost as compared to anterior defects, making their repair more challenging. In lateral abdominal wall defects, there is also a paucity of aponeurosis or fascia laterally for the abdominal muscles to be anchored to during a repair. This is in contrast to anterior abdominal wall defects where the linea alba and linea semilunaris can be easily utilized as stable points of fixation. Certain anterior abdominal wall defects such as ventral hernias consist only of a fascial defect as compared to lateral abdominal wall defects which always invariably involve a muscular component. Muscle is much harder to reconstruct compared to fascia due to its dynamic nature and thickness compared to fascia whose properties can be easily reproduced with synthetic or biologic meshes. Lateral defects are also asymmetrically located compared to anterior defects that are at the midline. The remnant ipsilateral rectus abdominis muscle and contralateral abdominal wall muscles continue to exert a pull with the lateral defect now the weakest point in the abdominal wall. Over time, the imbalance of forces predisposes to bulge and herniation at the repair site.

The fundamental principles of lateral abdominal wall reconstruction are similar to that of ventral hernia repair [3]. The goal is a strong and durable reconstruction, which is most effectively achieved by mesh-reinforced innervated musculofascial reapproximation [4].

This means that postresection or debridement of the pathology, healthy fascia, and muscle should be mobilized and repaired as much as possible to establish complete fascial closure, followed by mesh placement to achieve a sturdy dual-layer repair. This is not always attainable in lateral abdominal wall defects where the aponeurotic fascia is limited and there is no option of component separation to mobilize the muscle complexes as in ventral hernia repairs. In most cases, complete dual-layer repair cannot be obtained. Hence, secure mesh placement is very important in determining the strength and stability of the overall repair. Instead of patchwork repair which may give rise to areas of weakness over time due to muscular denervation, the entire lateral abdominal wall should be reinforced with mesh anchored to points located past the origins and insertions of the muscle complexes. Traditionally, mesh may be placed in an onlay, inlay, or sublay fashion. The terminology is most often used in relation to anterior abdominal wall defects. In onlay mesh repair, the mesh is placed in the subcutaneous space over the repaired anterior rectus fascia and anchored to it with sutures. It is technically easier to perform as compared to sublay mesh repair but is associated with higher rates of hernia recurrence, wound infection, and seroma formation [5]. Sublay mesh repair involves placing a mesh in between the closed posterior rectus sheath and the rectus abdominis muscle. The inlay technique is used when the fascial edges cannot be reapproximated and the mesh is placed to bridge the defect. In cases where the lateral abdominal wall defect is sizable and fascial closure cannot be achieved, inlay mesh placement with the mesh anchored to stable points of fixation such as innervated musculofascia, aponeurotic tissue, or bone is recommended. Superiorly, stable pillars for fixation include the ribs and the coastal margin. Inferiorly, the iliac crest and inguinal ligament may be utilized. Posteriorly, the mesh can be secured to the paraspinal fascia.

Desmoid tumours are the most common soft tissue tumour of the abdominal wall [6]. Although histologically benign and lacking metastatic potential, they are commonly locally invasive and require radical resection with full-thickness abdominal wall excision. Despite this, local recurrence rates are as high as 50% at the 5-year mark [7].

## 2. Case Report

**Figure 1:**
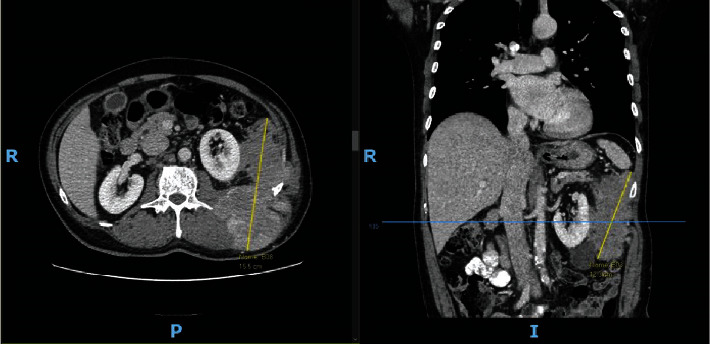
Preoperative CT scan of the patient. Note: CT scan showing an ill-defined mass in the left retroperitoneum and paracolic gutter, extending beyond the left posterior abdominal wall.

A 62-year-old Malay gentleman with hypertension, hyperlipidemia, and ischemic heart disease presented to our service with a 6-month history of a left upper back lump associated with loss of weight. Preoperative magnetic resonance imaging (MRI) showed an aggressive, infiltrative soft tissue tumour with involvement of the spleen, left kidney, pancreas, descending colon, ribs, and paravertebral muscles. Considerations included a primary aggressive sarcomatous tumour, lymphoma, or large metastatic deposit. The computer tomography (CT) scan of the thorax, abdomen, and pelvis of the patient is shown in Figure 1. It depicts an ill-defined mass in the left retroperitoneum and paracolic gutter, extending beyond the left posterior abdominal wall, measuring 15.5 cm by 12.3 cm. There were no distant metastases.

Biopsy showed a low to intermediate-grade spindle cell tumour, and the differentials were a liposarcoma or a desmoid tumour. The case was discussed at a multidisciplinary tumour board, and a decision was made for surgical excision with reconstruction.

**Figure 2:**
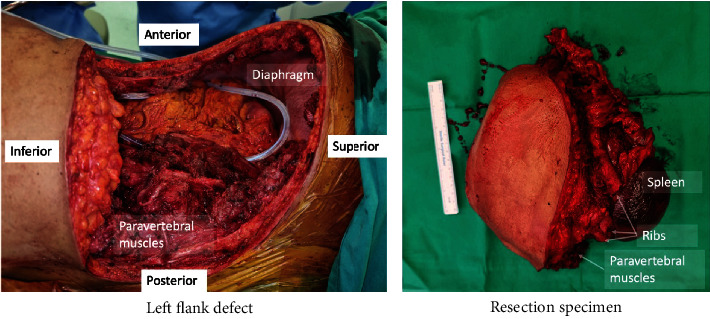
Clinical photograph of postresection. Note: resection specimen and resultant left flank defect.

The patient subsequently underwent resection of the tumour, left lower chest wall and ribs, left anterolateral thoracoabdominal wall, left lower lung lobe and diaphragm, limited left hemicolectomy, distal pancreatectomy, and splenectomy with left nephrectomy. The final specimen is shown in Figure 2. Resection extended superiorly to the 9^th^ rib, inferiorly to 3 cm from the iliac crest, posteriorly to the thoracolumbar transverse process, and anteriorly to the linea alba. The remnant diaphragm was repaired primarily with Vicryl 3/0 and sutured to the 8th rib. The resultant defect over the left flank measured 30 cm by 30 cm.

**Figure 3:**
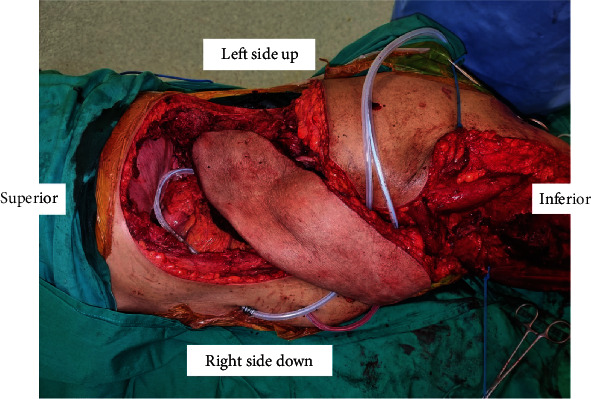
Clinical photograph of flap tunnelling. Note: ALT flap tunnelled under rectus femoris and sartorius then transposed superiorly and inset to left flank defect.

PROLENE® soft polypropylene mesh was stitched in an inlay fashion to the deep surface of the remaining chest and abdominal wall. Anteriorly, the mesh was anchored to the remaining linea alba. Superiorly, sutures were passed directly to the periosteum of the remaining 8^th^ rib. The mesh was stitched to the remaining fascia of the paravertebral muscles posteriorly and to the inguinal ligament inferiorly, forming a stable construct with 4 points of anchorage. To minimize bowel adhesion, the remaining omentum was mobilized and used to interface between the bowel and the synthetic mesh. The left extended anterolateral thigh (ALT) flap was raised in the subfascial plane with a large cuff of vastus lateralis and tensor fascia lata. A descending branch of the lateral circumflex femoral artery was identified and taken with muscle. The flap measured 28 cm by 14 cm and was tunnelled under the rectus femoris and sartorius then transposed superiorly for inset to the left flank defect as demonstrated in Figure 3.

**Figure 4:**
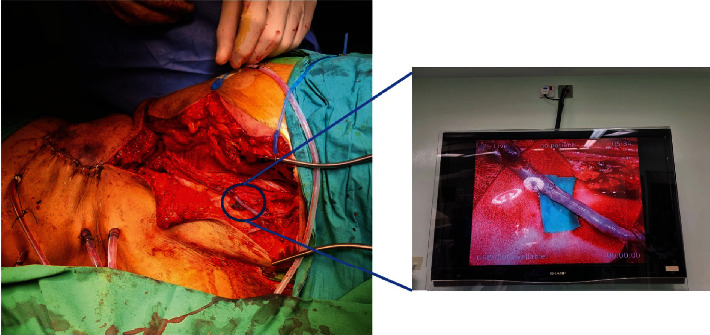
Clinical photograph of microanastomosis Note: GSV transposed superiorly and anastomosed end-to-end to the flap vein.

After partial flap inset, the flap was noted to be slightly purple in colour with dark bleeding on the pinprick. The pedicle was checked and not kinked in alignment. A decision was hence made to perform venous supercharging with an anastomosis to the great saphenous vein (GSV). The ipsilateral GSV was dissected, oriented upwards, and anastomosed end-to-end to the flap vein with Ethilon 9/0, as shown in Figure 4. Flap colour improved thereafter, and there were no further issues with venous congestion upon serial flap monitoring. The flap donor site was closed with a split skin graft.

One drain was placed to the left lateral of the stomach and another drain to the pelvis. These were sequentially removed on POD 17 and 21, respectively. Postoperatively, the patient was kept nil by mouth and covered with intravenous rocephin and flagyl for a week. He was nursed supine propped up 45 degrees and made to rest in bed for the first week postoperatively. Prevenar vacuum-assisted closure dressing was placed over the flap to assist with wound healing and removed on POD 7. A physiotherapist assisted the patient in bed exercises including knee and shoulder ranging and chest physiotherapy. He was advised to avoid stretching the trunk or applying pressure over the flap and to refrain from lying on his left side. From the second week onwards, the patient was allowed to sit over the edge of the bed and to transfer to a chair. At the third-week mark, he was able to ambulate with assistance. Initially, the patient was significantly deconditioned and relied heavily on a rollator frame. By POD 41, the patient was able to walk unassisted. The flap was monitored clinically using the Massimo transcutaneous oxygen monitoring system. Diet was slowly escalated, but the patient had significant ileus and had to be placed on total parenteral nutrition. Oral feeds were only commenced on POD 25 after a water-soluble contrast study showed normal intestinal transit time. The patient continued 3 weekly physiotherapy sessions postdischarge for two months. His main residual morbidity was mild tightness in his left thigh and left flank area. There was no weakness of hip flexion or knee flexion and extension with a power 5 on the MRC scale.

**Figure 5:**
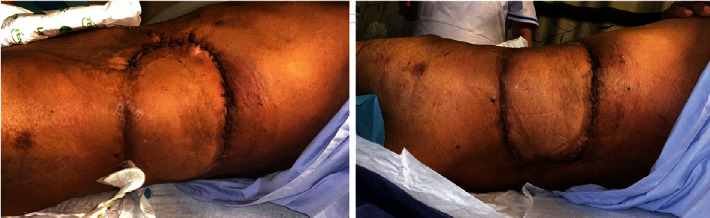
Clinical photograph of the patient on POD 40. Note: lateral and posterior views of the reconstruction on POD 40.

At clinic review on postoperative day (POD) 40, the surgical wounds were well healed and there was a good contour of the lateral abdominal wall, as shown in Figure 5.

**Figure 6:**
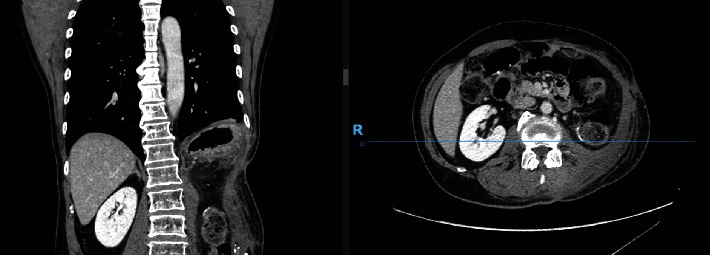
Postoperative CT scan of patient at 6-month mark. Note: CT scan of the lateral abdominal wall reconstruction at 6 months postoperatively.

**Figure 7:**
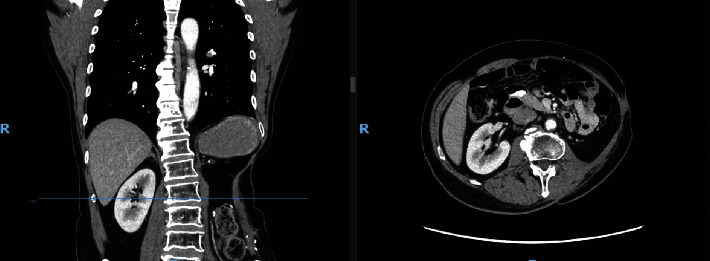
Postoperative CT scan of patient at 2-year mark. Note: CT scan of the lateral abdominal wall reconstruction at 2 years postoperatively.

Figures 6 and 7 show the surveillance CT scans at the 6-month and 2-year mark, respectively, demonstrating no recurrence of the tumour. There was also good integration of the mesh and flap with no hernia or bulge.

## 3. Discussion

The proximally pedicled ALT flap is a versatile flap with a long consistent pedicle, wide arc of rotation, and large potential size. The lateral arc has been shown to safely extend to the posterior superior iliac spine [8]. Despite this, most clinical case series feature the pedicled ALT flap for reconstruction of defects in the lower abdomen or suprapubic region [9]. In our case, there was sufficient pedicle length for rotation to the defect at the left flank. In cases where there is insufficient length, microsurgical pedicle lengthening may be performed using the muscular or transverse branches of the lateral circumflex femoral artery [10].

The pedicled ALT flap is known for its reliable blood supply, but there is a risk of venous congestion, particularly in distally based flaps. Venous augmentation has been shown to improve the reliability of distally based ALT flaps, and prophylactic venous supercharge with the GSV has been recommended to restore antegrade venous drainage in such flaps [11]. For this patient, there was venous congestion noted after partial flap inset which is rare for proximally based flaps. This could be due to the large size of the flap and the small calibre of the venae comitantes.

In our case, the ALT flap provided sufficient size and strength as a coverage option. While there was sufficient deep fascial tissue to anchor the mesh to, in cases where there is a paucity of such fixation points, drilling of suture holes through the inferior ribs or pubic bone may be necessary. In anterior abdominal defects, component separation can be performed to increase the available soft tissue coverage and offload tension on the repair. While this is not possible in lateral defects, we significantly reduced the defect size by raising a right abdominal wall skin flap of the muscle fascia and advancing it to the left, quilting it down with Stratafix. The discrepancy between the vertical height of the defect (30 cm) and the width of the flap (14 cm) was overcome by undermining the superior and inferior abdominal wall skin flaps and stretching the ALT flap. Despite this, the patient still had significant tightness on the reconstructed side. This gradually improved, and the patient did not develop any long-term truncal imbalance.

The final histology report returned as a desmoid tumour. The margins of resection were clear, but the posterior margin corresponding to the left paravertebral muscles was <1 mm. Hence, the patient underwent adjuvant radiotherapy to the left paravertebral muscles. In terms of functional recovery and quality of life, on outpatient clinic review at 2 months postinitial surgery, the patient was still using a walking stick and relied on his wife for assistance for his activities of daily living. He was mainly homebound and only went out to attend his clinic reviews and rehabilitation sessions. At this point, he was still attending regular physiotherapy sessions which focused on core and lower limb strength and balance retraining. At 6 months of outpatient clinic review, the patient had improved tremendously and was able to walk without aids. He began to gradually return to his former social activities including painting. At the 2-year follow-up visit after the index surgery, the patient was active and all his wounds were well healed with no incisional hernia noted on clinical examination. His surveillance scans did not suggest any local or distant recurrence.

We were concerned about how the repair would affect long-term abdominal wall function and core stability given the large size of the defect. To achieve musculofascial reapproximation, we harvested the extended ALT flap with the tensor fascia lata to bridge the gap in the fascia layer and correspondingly the vastus lateralis for the gap in the lateral abdominal muscle layer, thereby replacing like with like. Although a large cuff of vastus lateralis muscle was taken, reinnervation was not performed; hence, it was expected that the muscle would gradually atrophy with time which is evident on serial CT imaging that showed thinning of the muscle and replacement by subcutaneous fat. Fortunately, the patient was able to compensate with hypertrophy of the remaining abdominal muscles on the contralateral flank to counter-balance the area of weakness and did not exhibit any signs of truncal imbalance, being able to sit for long hours unsupported to paint.

The main limitation of our study is that it is a single case report and the results cannot be generalized. A cause and effect relationship also cannot be established between performing venous anastomosis and improvement of flap outcomes. Future research could include a case series or comparative cohort studies to evaluate various techniques of lateral abdominal wall reconstruction. A case series on the use of ALT flaps for lateral abdominal wall defects could also shed light on the dimension of the flap at which venous supercharging should be recommended.

## 4. Conclusion

The pedicled anterolateral thigh flap can be effectively used for the reconstruction of extensive lateral abdominal wall defects with a satisfactory functional and cosmetic outcome. Venous supercharging with the GSV can be performed in larger flaps with small-sized venae comitantes to reduce the incidence of venous congestion.

## Data Availability

The image and clinical data used to support the findings of this study are included within the article.
